# Developing a patient-centered outcome measure for complementary and alternative medicine therapies I: *defining content and format*

**DOI:** 10.1186/1472-6882-11-135

**Published:** 2011-12-29

**Authors:** Cheryl Ritenbaugh, Mimi Nichter, Mark A Nichter, Kimberly L Kelly, Colette M Sims, Iris R Bell, Heide M Castañeda, Charles R Elder, Mary S Koithan, Elizabeth G Sutherland, Marja J Verhoef, Sarah L Warber, Stephen J Coons

**Affiliations:** 1 Department of Family & Community Medicine, The University of Arizona, Tucson AZ, USA; 2 School of Anthropology, The University of Arizona, Tucson AZ, USA; 3 Department of Anthropology, University of South Florida, Tampa FL, USA; 4 Kaiser Permanente Center for Health Research, Portland OR, USA; 5 College of Nursing, The University of Arizona, Tucson AZ, USA; 6 National College of Natural Medicine, Portland OR, USA; 7 Department of Community Health Sciences, University of Calgary, Calgary, AB, Canada; 8 Department of Family Medicine, University of Michigan, Ann Arbor MI, USA; 9 Patient-Reported Outcome Consortium, Critical Path Institute, Tucson AZ, USA

**Keywords:** Complementary and alternative medicine (CAM), patient-reported outcomes (PROs), patient-centered care, non-specific outcomes, questionnaire development, retrospective pre-test, well-being

## Abstract

**Background:**

Patients receiving complementary and alternative medicine (CAM) therapies often report shifts in well-being that go beyond resolution of the original presenting symptoms. We undertook a research program to develop and evaluate a patient-centered outcome measure to assess the multidimensional impacts of CAM therapies, utilizing a novel mixed methods approach that relied upon techniques from the fields of anthropology and psychometrics. This tool would have broad applicability, both for CAM practitioners to measure shifts in patients' states following treatments, and conventional clinical trial researchers needing validated outcome measures. The US Food and Drug Administration has highlighted the importance of valid and reliable measurement of patient-reported outcomes in the evaluation of conventional medical products. Here we describe Phase I of our research program, the iterative process of content identification, item development and refinement, and response format selection. Cognitive interviews and psychometric evaluation are reported separately.

**Methods:**

From a database of patient interviews (n = 177) from six diverse CAM studies, 150 interviews were identified for secondary analysis in which individuals spontaneously discussed unexpected changes associated with CAM. Using ATLAS.ti, we identified common themes and language to inform questionnaire item content and wording. Respondents' language was often richly textured, but item development required a stripping down of language to extract essential meaning and minimize potential comprehension barriers across populations. Through an evocative card sort interview process, we identified those items most widely applicable and covering standard psychometric domains. We developed, pilot-tested, and refined the format, yielding a questionnaire for cognitive interviews and psychometric evaluation.

**Results:**

The resulting questionnaire contained 18 items, in visual analog scale format, in which each line was anchored by the positive and negative extremes relevant to the experiential domain. Because of frequent informant allusions to response set shifts from before to after CAM therapies, we chose a retrospective pretest format. Items cover physical, emotional, cognitive, social, spiritual, and whole person domains.

**Conclusions:**

This paper reports the success of a novel approach to the development of outcome instruments, in which items are extracted from patients' words instead of being distilled from pre-existing theory. The resulting instrument, focused on measuring shifts in patients' perceptions of health and well-being along pre-specified axes, is undergoing continued testing, and is available for use by cooperating investigators.

## Background

Complementary and alternative medicine (CAM) systems are widely used among individuals who continue to use conventional medicine [1]. CAM encompasses healing systems such as traditional Chinese medicine, acupuncture, naturopathy, homeopathy, chiropractic, Ayurveda, massage therapy, yoga, tai chi [2], and eclectic blends of health practices [3]. Most CAM practitioners seek to promote well-being in the "whole person" as much as reducing specific symptoms that the patient may be experiencing as signs of larger underlying problems [4-8]. Multiple studies report that as a result of CAM therapies, many patients experience shifts in well-being that extend beyond resolution of the "presenting" symptoms [4,8-18]. Reported shifts include improvements in overall well-being, energy, clarity of thought, emotional, social, and physical functioning, and increased focus on one's inner life and spirituality [4,5,7,9]. Shifts in one domain of life are often reported to be linked to other positive lifestyle changes; for example, a mind-body intervention may foster adherence to beneficial lifestyle changes [11].

CAM practitioners participating in research have expressed a need for more appropriate measurement tools that capture the multiple diverse shifts in patients' states following treatment [6]. Numerous specific measures and scales have been applied in the assessment of CAM interventions to date (e.g. pain, fatigue, fibromyalgia); however, most of these scales were developed for use in the study of conventional therapies. What has not been available is an instrument developed from the perspective of the CAM user that would measure the most common and important shifts in well-being that they experience [6,12,19,20]. The development of measurement tools for evaluating CAM therapies has to date not been based on qualitative data relating to the range of subjective experiences that patients recognize as outcomes of therapeutic interventions. The closest measure [21,22] used patient and practitioner input, but began the process with a 100-item list drawn from existing quality of life scales, thus orienting the participants to existing constructs from the start rather than relying on them to provide their unfiltered experience.

The goal of our research program was to develop a measurement tool with acceptable participant burden that could be used to systematically assess a variety of shifts in well-being across a broad range of therapeutic modalities and conditions. We hoped that the resulting instrument would be sufficiently complete to minimize the need for those using it in their clinical practice and/or research studies to restrict themselves to a narrow set of outcome domains. The multiple phases of the project, including both the secondary analysis of people's experiences and the new data presented in this paper, have allowed us to identify a set of what have often been called 'non-specific' outcomes of CAM therapies.

Along with others [20,23,24], we argue that it is no longer appropriate to label these outcomes 'non-specific' when, as we show here, they can not only be identified, but also captured by a standardized instrument that is patient-centered and derived from their actual experiences. Further, these multidimensional outcomes are integral to the practice theories and clinical predictions of the major CAM systems. For instance, Traditional Chinese Medicine (TCM), classical homeopathy, and Ayurveda utilize constitutional diagnostic procedures with integrative assessments of the patient as a complex interconnected network, as well as treatment plans intended to normalize the diagnosed person-wide disturbance that underlies the multi-system symptom pattern [25,26]. Therefore, we use the broad term 'emergent outcomes' to refer to those seemingly indirect outcomes that may be beyond the direct biomedical endpoints for which patients sought therapy, and may or may not have been part of the expected outcomes from the perspective of the CAM practitioners [20,23,24].

In creating such an instrument, we have recognized the need to be attentive to both multi-dimensionality and multi-directionality of shifts. For example, cancer patients may experience a decline in physical health while reporting a concurrent improvement in their sense of well-being. In addition, individuals with less life-threatening conditions may experience a temporary sense of discomfort or disease preceding a shift to a new subjective state of being [27]. We further recognized that any new measurement instrument would need to assess changes in well-being that have positive valence rather than simply signifying the absence or reduction of negative states. This follows the lead taken by positive psychology, which has shifted the focus from mental illness to mental health [28-30].

### Patient-Reported Outcomes

The need for a new type of outcome measure has also been identified in conventional medical research by the emergence over the past decade of the term *patient-reported outcomes *(PROs). PROs can be described as the consequences of ill health and/or its treatment as reported by patients, including perceptions of health, functioning, well-being, symptom experience, side effects, and treatment satisfaction. The importance of the appropriate measurement of PROs in clinical trials was underscored by the release of the US Food and Drug Administration's (FDA's) guidance for industry titled *Patient-Reported Outcome Measures: Use in Medical Product Development to Support Labeling Claims *[31]. As stated in the guidance, "Use of a PRO instrument is advised when measuring a concept best known by the patient or best measured from the patient perspective." The intent of the guidance was to describe how the FDA will evaluate the appropriateness and adequacy of PRO measures used as effectiveness endpoints in clinical trials. PRO endpoints are increasingly being used to complement conventional indicators of treatment benefit (e.g., clinician-reported outcomes, biomarkers) in trials [32]. They inform and enrich the evaluation of therapeutic interventions by providing the patient's perspective and, in some cases (e.g. pain), a PRO may be the only feasible endpoint in a clinical trial because there are no observable or measurable physiological markers of disease or treatment activity [33].

To the best of our knowledge, there has been no previous attempt to create a PRO instrument that captures the emergent outcomes of CAM therapies as described above. Most PRO instruments developed for use in clinical trials are aimed at assessing the specific symptoms (e.g., pain, nausea, itching) or aspects of functioning (e.g., joint stiffness, shortness of breath on exertion) that are the primary target of the intervention being evaluated. Nevertheless, the emergent outcomes that may occur independent of symptom relief and enhanced physical functioning are relevant and legitimate PROs that warrant measurement in valid and reliable ways. While our research has been largely informed by the PRO literature, we have chosen to use the term 'patient-centered' rather than 'patient-reported' in the title of this paper in order to denote that our work is the result of an in-depth process which puts the patient and his/her experience at the center of the process of identifying and determining meaning for emergent outcomes.

Scientists attempting to prospectively and systematically measure emergent outcomes in their CAM clinical trials are faced with the dilemma of not knowing which of the many such outcomes to target, but having to identify in advance a small number of endpoints, since available measurement instruments are often narrowly focused on individual domains or concepts (e.g., fatigue, affect, resilience). The Canadian Interdisciplinary Network for Complementary and Alternative Medicine Research (IN-CAM) responded to the need of CAM investigators for identification and access to instruments by developing a database that summarizes and categories existing outcomes measures http://www.outcomesdatabase.org. However, this does not address the issue that a battery combining individual PRO instruments can become quite large and cumbersome, resulting in unacceptable levels of respondent burden.

The guiding premise of our work has been that the patient's perception of personal changes associated with a CAM intervention is one of the most relevant measures of its impact. In this paper, we report on this mixed methods approach to develop outcome measures for CAM therapies. Medical anthropology has long been interested in subjective states of illness and healing, but to date anthropologists have not actively participated in the development of instruments systematically designed to capture these states for purposes other than description. Here, we iteratively combined qualitative ethnographic and psychometric methods to identify emergent outcomes to be measured, and to develop tools for that measurement. Phase I, reported here, details the iterative process of content identification, development, and refinement of items that capture patient-centered outcomes associated with CAM. Phase II, the quantitative and qualitative validation component, is reported in separate papers.

Rather than starting with an initial item pool based on expert panels or existing instruments, our content identification phase began with a secondary analysis of in-depth interviews with CAM patients collected during previous projects. Relevant language from these studies (described below), including words and phrases used by patients to describe emergent outcomes following CAM therapies, were identified to enable creation of an instrument from the "bottom-up." To further enrich this pool of subjective accounts and to identify a robust, minimal set of terms that could be endorsed by the maximal number of people, we undertook further interviews and analysis with the goal of identifying the content and format of a preliminary patient-centered outcome measure intended for use in clinical trials of CAM, as well as by CAM and other practitioners in their private practices.

## Methods and Results

Phase I of the project consisted of three research activities: content generation, item reduction, and format development. The first (Phase Ia) entailed the mining of preexisting qualitative data sets to generate an item content pool (see Table 1 for study details) [13,14,18,34-36]. The second (Phase Ib), involved further evaluation, refinement, and reduction of that item pool through evocative card sort interviews. Because the results of the first research activity were the basis for the second activity, we present the methods and results from each separately and sequentially. The third activity was the identification and development of an appropriate format to be used in the measure (Phase Ic), which occurred simultaneously with the other two.

**Table 1:** Descriptions of original data sets

	Study Title and CAM therapies involved	Study sample	Setting	Objective	Design
1	*Patient perspectives on homeopathic treatment *[13]	42 classical homeopathic patients (31 women, 11 men ages 22-73)	Wide range of homeopathic clinics	To describe the lived experience and outcomes of successful homeopathic treatment	In-depth individual patient interviews (47 interviews)

2	*Forgoing conventional treatment and using (a wide variety of) complementary therapies by men with prostate cancer *[18]	29 men with prostate cancer (aged 50-82) who forgo all conventional treatment in favor of alternative treatment	No specific setting: eligible men responded to recruitment posters	To examine which factors influence men with prostate cancer to decline all conventional cancer treatment, and learn about their experiences.	27 in-depth qualitative interviews with 11 individuals.

3	*The impact of Healing Touch on headache *[35]	13 headache patients (10 women, 3 men; ages 25-61) who received energy medicine treatment	Specialty pain clinic @ group model HMO	To document the range of complex, multi-dimensional outcomes possible with CAM therapies; to identify concepts and language that capture an individual's explanatory model of healing	In-depth individual patient interviews (29 interviews)

4	*Alternative medicine (naturopathy and TCM) approaches for women with temporomandibular dysfunction (TMD) *[34]	16 women (ages 25-55) with temporomandibular dysfunction and several other health disorders (10% sample from study n = 150)	Specialty TMD clinic @ group model HMO	Explore experiences with treatments and practitioners; discuss outcomes of treatment	Individual interviews with a subset of participants in a Phase II RCT (16 interviews)

5	*Supporting the transformative process: experiences of cancer patients receiving integrative care *[14]	11 cancer and HIV/AIDS patients (5 men and 6 women, aged 35-70) seeking integrative care	Integrative clinics in Vancouver BC	To describe essential features of the transformative experience among people living with cancer who seek integrative care; to identify factors supporting this process	In-depth individual patient interviews (16 interviews)

6	*Experiences of CVD patients encountering (a wide variety of) CAM therapies *[36]	26 participants (14 male, 12 female; ages 43-80) with cardiovascular disease	Newspaper ads and flyers looking for CVD patients who had experienced CAM therapies	Explore patients' experiences of CAM therapies in relation to their experiences with heart disease	A total of 15 open-ended interviews were completed (12 individual; 3 group)

### Phase Ia: Secondary Analyses of Existing Qualitative Data Sets

#### Ia: Methods

In Phase Ia of the project, we utilized patient transcripts (n = 177) from six peer-reviewed externally funded studies of the outcomes of CAM therapies conducted between 2001 and 2004 [13,14,18,34-36]. While most of the interview data from these projects were not collected for the purpose of identifying shifts in well-being following CAM treatment, the transcripts provided a rich source of data on patient-reported experiences with CAM therapies including subjective accounts of treatment effect. The six CAM studies involved a broad range of study designs, clinical sites, CAM interventions, and disease states, summarized in Table 1. Quality criteria (typically identified as reliability) in qualitative research relates to efforts by researchers to assure faithful and credible representation of reality as observed or studied [37-39]. All six original studies used several acceptable methods to increase the credibility, including respondent validation, audibility of data collection and analysis procedures, negative and deviant case analysis, triangulation across multiple researchers for each study, and close adherence to the *emic *(the subjects' own language and representations) perspective in the creation and reporting of outcomes.

A coding team at each of the six institutions where the data were originally collected completed transcript analysis. The lead analysis team located at the University of Arizona conducted weekly teleconferences with coders from all sites. A password-secured server was set up for the exchange of files, with only short excerpts from interviews shared across sites to protect participant anonymity. The University of Arizona Institutional Review Board (IRB) and other relevant institutional IRBs approved all procedures.

As a first step, the coding team reviewed all available transcripts with the goal of selecting those that had content related to shifts in well-being which could be used for further analysis. The research teams (investigators and staff from each site) met by telephone conference call to achieve consensus on the shifts in well-being that would make a transcript eligible for secondary analysis, and to establish the overall parameters by which they would proceed with coding. These parameters included (1) biopsychosocial (i.e. physical, psychological, social, spiritual) outcomes experienced by the participants that were beyond changes to chief complaints and (2) changes in consciousness or life experiences described by the participants that patients attributed to the CAM modalities studied.

This resulted in the selection of 150 interview transcripts from 119 individuals in which participants spontaneously discussed shifts in well-being associated with CAM treatment. In the next step, a codebook was developed to facilitate the identification of dimensions of change. The coding utilized both deductively derived codes identified by the research team and informed by their understanding of previous studies, and inductively derived codes that emerged from the data and reflected the language of the participants. Initial codes were established in consultation with the entire research team by identifying the larger themes found in transcripts across the different studies. The coding team then used these initial codes to tag transcript segments. Coding was aided by the use of ATLAS.ti version 5.2 http://www.atlasti.com qualitative data analysis software.

As coding progressed, initial broad themes were refined. For example, the original theme of "engaging in life differently" was adjusted to capture more specific features that appeared upon a close reading of the transcripts, with coding moving to specify "lifestyle changes" or "attitude changes." All emergent codes were discussed during weekly analysis team meetings and added to the codebook, when appropriate. Segments with specific codes were compared across sites during weekly meetings to ensure inter-rater reliability. In cases where codes were used differently across sites, codebook definitions were carefully recalibrated, and coders recoded their data to ensure consistency. In this process, close attention was paid to the words and phrases used by participants to describe shifts they experienced. Once the themes were identified from the transcripts, a "conceptual translation" process was employed to move toward items that could be included in a measurement instrument that was intended for wide use. This process essentially moved from the evocative and often metaphorical language of the patient to a more general and widely meaningful patient-centered outcome. Examples of the metaphorical language of quotes and the derived draft items are presented in section Ia: Results below. We attempted to neutralize local or regional language, CAM-therapy-specific language, and gender-specific language.

#### Ia: Results

We generated a relatively large and rich pool of candidate items from this analysis, including items relating to states of "unwellness," the experiences of transitional states and processes, and states of greater well-being. Examples of the metaphorical language from the original interview transcripts, and sample simplifications, are shown in Table 2. This list of items was then shared with CAM practitioners (n = 30) who had previously participated in research studies (see Table 3 for a description of provider demographic and practice characteristics). They were asked to review and add to the pool any additional items that patients in their practices often reported, including descriptors of both negative and positive states. Items added by practitioners at this stage tended to focus on physical functioning, and included sleep, physical symptoms, slow/fast recovery, and "bouncing back."

**Table 2:** Outcome domains, representative quotations, and associated simplified item content for components of change.

Outcome domains	Quotations (Examples of metaphorical expressions)	Items (for questionnaire)
**Physical**	"I would just take Ibuprofen because it would be so painful. You would have to go to work and stuff. When I first had it I couldn't even go to work." (4) "A big part of it is just feeling so exhausted and tired and just like not, you know I'm fairly able to function but I'm like sitting there at work. I'm functioning but I'm not focusing. My mind isn't clear and... [Talks to kids in background]. Like I'll be, just my inner energy, that's the word. My energy has just dissipated. You know, like I'll be in bed, by the time I get home I'll be in bed. I'll sleep. I'll wake up exhausted. I'm just, I just feel drained." (1) "It's like my inside furnace is working better for me. Not like when I was healthy, it's a totally different furnace. I had to rebuild it; the old one broke" (3) "I've just learned to pick up on signals that I know when I'm completely in synch and I'm able to handle the stress load, which is not going to change for me any time soon." (5) "I'm going to do very well at this. I'm going to amaze the doctor with my speed of healing and my range of motion or my strength....he called me impressive." (6)	*I was in pain*. *I felt drained*. *I was tired/I had no energy/I was exhausted*. *I felt depleted*. *I didn't sleep well*. *I am in tune with my body*. *My body recovers quickly*.

**Social**	"It got to be very isolating and very lonely. I cried a lot. Always. I always cried a lot over nothing, I'd just all of a sudden start crying for no reason. That whole feeling of worthlessness, you know, and not being able to have healthy relationships with people because I was so, I didn't feel like I was deserving of that. Very lonely. Just kind of like everyone would be better off without me and always thinking that way and always thinking about leaving or running away." (1) "This has been a phenomenal thing for me. This was a need that became more in focus. I had an overwhelming desire to help people with this gift that had manifested in me, that drew me to be more open with people." (3)	*I felt alone*. *I feel connected*.

**Psychological -- Cognitive**	"It's very hard to, um, sort of live life normally, because you're going to blood tests, you're going to counseling, first 6 months you're in a fog, um, my work productivity went down by 60%. Most days, I'd just sit looking out the window surfing the net, or if I had time, I couldn't really focus too much." (5) "Part of me that was so deep that I couldn't even think...it wasn't a thought, it was just felt in every cell...that finally that I was seeing what I needed to see." (1) "I would say, or people would say about me, that I'm more compassionate with myself and others. But more important with myself. I mean that cancer ridden, cancer woman. I'm gentler, I'm softer. More forgiving, um, I don't have other words to describe it." (1) "The internal dialogue is changing, I'm not so hard on myself, I don't beat myself up so often." (3) "I think I learned to really like myself a lot more." (4)	*I was unable to focus*. *I couldn't think clearly*. *I am forgiving*. *I have learned new things about myself*. *I feel empowered*.

**Psychological --Affective**	"Well, sometimes having trouble sleeping, you know, waking frequently, and just feeling unnerved more than usual, more anxious, and, of course, then if I take my blood pressure and it's up then I feel even more anxious!" (6) "For I'd say 6 months I was in a very depressed way. Everything looked black. I'm very, as a rule, a positive person but everything looked very bleak, black and gray." (1) "I'm happy again. I'm laughing again. It's like, wonderful." (5) "I'm really satisfied and content." (5) "I feel more lighthearted like I can just laugh and play instead of always being worried about stuff." (5)	*I was anxious about the future*. *I was depressed*. *I laugh*. *I am content*. *I am joyful*.

**Spiritual**	"I felt so hopeless before. I never was actively suicidal, but I, I remember not caring. Just sort of thinking, well if I could just go to sleep and never wake up, that would be better, I'm just consuming a lot of resources." (6) "As I said before I really, really had a very, very strong sort of intuitive sense that this illness is not - it's a spiritual journey and it has been incredibly wonderful actually." (2) "All of a sudden one day I found that there was a spiritual feeling inside. It was not religious, it was spiritual. It was a wonderful feeling. It changed my life, and I still experience it." (1)	*I had no hope*. *I am on a spiritual path*. *I feel spiritual*.

**Whole person**	"I was always in crisis. I was in crisis about the relationship I was in. I was in crisis because I wasn't sleeping. I was in crisis because I didn't want to eat. I was in crisis because I was eating. I was in crisis because I was losing weight. I was in crisis because I couldn't hold a job. I mean it was just, it did not matter where I looked or what I did or, yeah, it was, my life was a mess." (1) "I was just kind of spinning my wheels, spinning my wheels and all that kind of stuff." (5) "I just feel more grounded and I feel more complete. Not like so much a superficial thing but a deep down caring. Beyond scratching the surface." (1) "The other life was the life before and there was no other life and I had to create a whole new life. I'm telling you it's like somebody that woke up from a coma." (5) "I know I am prepared to handle whatever comes my way...I am more aware, of how I feel, of how to tweak this, to tweak that... to me healing is more about the spirit and freedom that comes from the reality that is within me." (1)	*My life was a mess*. *I just kept doing the same thing over and over*. *I was really stuck in some parts of my life*. *I feel more complete*. *I am awake*. *I am aware* *I'm living my life to the fullest*.

*Numbers in this table correspond to the studies in Table 1.

**Table 3:** Practitioner Characteristics for Item Development

Female	27 (90%)
Male	3 (10%)
**Race/Ethnicity**	
White, non-Hispanic	18 (60%)
African American	9 (30%)
Asian	1 (3%)
Hispanic/Latino	2 (7%)

CAM Therapy Practiced*	
Massage Therapy	7 (23%)
Naturopathy	4 (13%)
Traditional Chinese Medicine	4 (13%)
Reiki	2 (7%)
Holistic Health Education	2 (7%)
Midwifery	2 (7%)
Integrative Medicine	2 (7%)
Meditation	1 (3%)
Chiropractic	1 (3%)
Dietician/Nutritionist	1 (3%)
Biomagnetic Touch	1 (3%)
Homeopathy	1 (3%)
Qi Gong	1 (3%)
Soul Memory Discovery	1 (3%)
Personal Development and Literacy	1 (3%)
Ho'oponopono	1 (3%)

**Number of CAM Therapies Practiced**	
Mean (SD)	2 (1.67)

* Percentage based on the total number of providers (N = 30); providers were asked to list all the types of modalities they practice.

From these data sources, we created a filtered list of relatively broad terms that captured the meanings of a range of words and phrases. At a two-day all investigator meeting, these items were further categorized into five areas of health and well-being (physical, emotional/affective, cognitive, social, and spiritual) to identify their distribution across these frequently used psychometric domains. In the process of categorization, we discovered a sixth domain that we termed "whole person" for items that seemed to bridge several domains. The resulting item pool and assigned categories generated through Phase Ia are shown in Table 4 in the left hand column (the numerical rankings in this table are described below in section 1b Results: Quantitative Analysis).

**Table 4:** Complete List of Positive and Negative Items by Level of Endorsement* Sorted by Psychometric Domains

Item**	# of Applies or Best Applies (max n = 34)
**Whole Person Negative**	

I felt drained. (W - Phy - E)	20

I was overwhelmed. (W)	19

I was in pain. (W -- Phy)	18

I felt unbalanced. (W)	15

I was suffering (W)	15

I muddled through my days. (W)	14

I felt powerless. (W - Emp)	13

Every day was a struggle. (W)	13

I was miserable (W-A)	12

I was really stuck in some parts of my life. (W)	12

I felt numb. (W - Phy)	12

I felt shattered. (W - A)	11

I put all my energy into survival. (W - Phy)*	11

I was judgmental. (W)	10

I felt like I was sinking in quick sand. (W)	10

I felt alone. (W - So - Sp)	10

I was hanging on for dear life. (W - A)	10

My life was a mess. (W)	10

I was scared that my life might never get better. (W -- A)	8

I felt out of control. (W - Emp)	8

I felt defined by my illness. (W)	7

I just kept doing the same thing over and over. (W)	7

I was desperate. (W - A)	7

I lost myself. (W)	6

I didn't bounce back. (W)	6

Nothing gave me pleasure. (W)	5

There was no laughter in my life. (W - A)	5

I lost trust in my body. (W - Phy)	5

I was unforgiving. (W)	5

I felt like I was being punished. (W - Sp)	4

I was not resilient. (W)	3

I had tried everything and nothing worked. (W - Phy)	2

**Whole Person Positive**	

I am on a path towards health and wellness. (W -Phy)	22

My life has meaning. (W)	20

I take life's obstacles in stride. (W)	20

I am forgiving. (W)	20

I am able to forgive. (W)	20

I feel alive. (W - E)	19

I am able to let go. (W)	19

I am resilient. (W)	19

I feel empowered. (W - Emp)	19

I'm trying things I never tried before. (W)	18

I am awake. (W -- E)	17

I am aware. (W - Cog)	16

I feel vitalized. (W - E)	15

I am in control of things that I can control. (W - Emp)	15

My life is balanced. (W)	14

I am restored. (W - E)	14

I bounce back. (W)	13

I trust my body. (W -- Phy)	13

I'm feeling great. (W)	13

I am living my life to the fullest. (W)	13

I am nonjudgmental. (W)	10

Life is good. (W)	10

I feel complete. (W)	9

Life is not a struggle. (W)	9

**Physical Negative**	

I was tired/I had no energy/I was exhausted. (Phy - E)	20

I was depleted. (Phy - E)	18

I didn't sleep well. (Phy)	17

My senses felt dull. (Phy)	16

I have had improvements in my health I did not expect. (W - Phy)	15

I kept getting the same kinds of symptoms. (Phy)	13

It felt like my body was breaking down. (Phy)	12

My body recovered slowly. (Phy - Fxn)	8

**Physical Positive**	

My body recovers quickly (Phy - Fxn)	22

I am in tune with my body. (Phy)	18

I am better able to carry out daily activities. (Phy - Fxn)	18

My senses are vibrant. (Phy)	16

I sleep well. (Phy - E)	13

I do things now I hadn't been able to do before the [treatment/study] began. (Phy - Fxn)	12

I wake up rested and refreshed. (Phy - E)	11

I have abundant energy. (Phy - E)	6

I don't have symptoms any more. (Phy)	3

I have abundant energy to do what I want to do. (Phy - E - Fxn)	1

**Cognitive Negative**	

I couldn't think clearly. (Cog)	15

I was unable to focus. (Cog - W)	13

I felt like nothing I could do would help. (Cog - Emp)	7

**Cognitive Positive**	

I have learned new things about myself. (Cog - Sp)	21

I am able to deal with life's difficulties. (Cog)	20

I am better able to make decisions about my health and well-being. (Cog)	20

I am focused. (Cog)	17

My life is not defined by my illness. (Cog)	15

**Emotional/Affective Negative**	

I was stressed. (A)	20

I was scared. (A)	18

I am not afraid of the future. (A)	14

I was depressed. (A)	13

I was anxious about the future. (A)	11

I had an overwhelming sense of loss. (A)	11

I was so angry. (A)	9

Life was not worth living. (A)	8

All I could see was what I had lost. (A)	5

**Emotional/Affective Positive**	

I am able to love. (A - So)	21

I laugh. (A)	21

I find pleasure in life. (A)	21

I am joyful. (A)	21

I am happy. (A)	19

I am content. (A)	17

**Spiritual Negative**	

I had no hope. (Sp)	7

I felt far from God. (Sp)	6

I had lost my faith. (Sp)	4

**Spiritual Positive**	

I am using my inner resources to heal myself. (Sp)	21

I am hopeful. (Sp)	21

I have faith. (Sp)	20

I have hope. (Sp)	20

I feel spiritual. (Sp)	20

I am on a spiritual path. (Sp)	18

I feel closer to God. (Sp)	13

**Social Negative**	

I couldn't/wouldn't take suggestions from others. (So)	8

**Social Positive**	

I feel connected. (So - Sp)	18

* "Endorsement" means categorizing the item as "applies" or "best applies."

** Abbreviations in parentheses after each item reflect the following domains:

W = Whole Person; Phy = Physical; Emp = Empowerment; E = Energy; Fxn = Functional;

Sp = Spiritual; Cog = Cognitive; A = Emotional/Affective; So = Social

### Phase Ib: Evocative Card Sort Interviews

#### Ib: Methods

In order to test the fit of the list of shortened positive and negative phrases generated in Phase Ia to informant experiences of personal change and to capture other possible descriptors of positive and negative states, we created an innovative interview protocol to be used with a new pool of informants. Our goals with this phase were to identify a much shorter but widely endorsed set of markers of subjective states, and to obtain direct feedback on the wording of individual items (Table 4). An interview protocol was developed specifically to encourage informants to reflect on their states prior to and following CAM therapies, without requiring attribution of any changes to the therapies, and to select words and phrases which best captured their ranges of personal experiences.

We termed our interview strategy an "evocative card sort interview" in that it attempted to evoke both denotative and connotative meanings associated with words. Denotative language employs words or phrases to refer or point to a specific state or quality, such as a definitive symptom of an illness like fever or fatigue. Connotative language indexes a cluster of loosely associated images, schema and feelings about an experience that is particularly salient to an individual. For example, saying that one's energy has changed following a CAM treatment would be an example of connotative speech indexing a set of associations and feeling states. To the extent possible, we wanted to identify terms that captured widely endorsed evocative states, which were not highly idiosyncratic or culturally specific. We also wanted to identify descriptors that were scalable; that is, easy for many people to identify with as registers of change. We chose a "card sort" approach to interviewing subjects about their outcomes [40]. This was predicated on the recognition that for some individuals, their subjective shift may not have previously been articulated; that is, it may have been sensed internally but remained pre-verbal or pre-cognitive. Therefore, card prompts were used to trigger tacit knowledge and embodied memories as well as to provide frames of reference for experienced but thus far unspoken shifts in well-being.

The informants for this phase were recruited using a purposive sample approach at three of the sites that had been involved in Phase Ia of the project (Tucson, AZ, Portland, OR, and Vancouver, BC). Participants were recruited from two wellness centers frequented by cancer and HIV patients, from clinics, and from ads placed in local health magazines. We also asked CAM practitioners to refer patients to participate in interviews if they had reported significant shifts in well-being associated with CAM therapies (as defined above), as it would not benefit this part of the process to interview individuals who had not changed. We were careful to recruit a diverse set of individuals across multiple CAM systems and health conditions, as we were particularly interested in testing the relevance of the items for use with patients from a wide range of CAM therapies. After obtaining consent from individuals to participate in the interview, a letter was sent out prior to the interview asking the person to select a shift in well-being they had experienced following a CAM therapy and which they would be willing to share with the interviewer. Characteristics of the 34 participants are described in Table 5.

**Table 5:** Characteristics of Participants in Evocative Card Sort Interviews, Phase Ib

Age	
Mean (SD)	56.04 (11.5)
Range	30-81
**Gender**	
Female	28 (82%)
Male	6 (18%)

**Race/Ethnicity**	
White, non-Hispanic	20 (59%)
African American/Black	8 (24%)
Asian	4 (12%)
Hispanic/Latino	1 (3%)
Native American/Indigenous	1 (3%)

**Highest Level of Education**	
Some College, Associates Degree, or Technical Training	5 (15%)
Four-Year College Degree	4 (12%)
Postgraduate Coursework or Degree	17 (50%)
Missing	8 (24%)

**Reason for Using CAM Therapy**	
Pain	9 (26%)
Cancer	8 (24%)
Psychological/Spiritual Crisis	7 (21%)
HIV	3 (9%)
Grief	2 (6%)
Extreme Stress and Anxiety	2 (6%)
Addiction	1 (3%)
Trauma	1 (3%)
Hepatitis C	1 (3%)

CAM Therapy Used*	
Acupuncture	6 (18%)
Meditation	6 (18%)
Yoga	5 (15%)
Chiropractic	4 (12%)
Integrative Medicine	3 (9%)
Massage Therapy	3 (9%)
Naturopathy	2 (6%)
Diet/Nutrition	2 (6%)
Biomagnetic Touch	2 (6%)
Support Groups	2 (6%)
Traditional Chinese Medicine	2 (6%)
Herbal Medicine	2 (6%)
Homeopathy	1 (3%)
Tai Chi	1 (3%)

**Number of CAM Therapies Used**	
Mean (SD)	1.74 (.994)

Participants could report more than one therapy. Percentage based on the total number of patients (N = 34).

Because this interview protocol was innovative, interviewers required training in the card sort methodology. Each interviewer conducted four pilot interviews with people known to the research team using the evocative card sort method, thus providing them an opportunity to learn to work with the method and sensitizing them to how individuals might respond to the interview process. Interviewers were trained to allow informants sufficient time to "try on" the terms/phrases on the cards to determine if they fit their experiences. Importantly, interviewers were encouraged to be empathetic witnesses of the process.

At the onset of the interview, the interviewer explained that she was particularly interested in two stages that people encountered during the healing process: first, being in a tough spot (physically, emotionally, psychosocially, or spiritually), and second, a subsequent better place. Informants confirmed that they had this type of experience and were asked to share a specific story, both verbally and briefly in writing. If they subsequently shifted to another story while going through the cards, the interviewer would gently bring them back to the index event noted on the card as a form of an anchor.

The evocative card sort interview began by asking the informant to first reflect on the tough spot they had experienced. The interviewer presented the informant with 54 cards that contained short words/phrases derived from Phase Ia (shown in Table 4). Examples include "I was tired," "I felt betrayed by my body," "I was hopeless," "I felt out of control," "I felt vulnerable," and "I couldn't think clearly." The informant was instructed to go through the 54 cards and divide these largely negative descriptor cards into 3 stacks: "Applies to me (i.e., fits my experience)," "Not quite right," and "Does not apply." After the informant sorted the 54 cards, the interviewer reviewed the "not quite right" stack and asked the informant to suggest a modification of the item, if possible. Once modified, the informant was asked whether the item was then applicable to his/her experience and to place it in the appropriate stack (applies to me/does not apply). Next, the "applies to me" cards were sorted into domains by the interviewer, as a next step in further winnowing down the card choice. The interviewer picked up the selected cards in a particular domain and said: "These cards appear similar--which one(s) best describe your experience?" (e.g., cognitive domain: "I was unable to focus," or "I couldn't think clearly"). Some informants were able to identify a single card that best captured their experience, while others were unable to do so and viewed several cards as equally significant. Informants were also invited to alter the words on the cards to better fit their experience or to offer new words or phrases on blank cards. Few interviewees volunteered additional descriptors, suggesting that the list generated in Phase Ia provided reasonable coverage of the range of experiences.

When the card sort and ensuing discussion were complete, the interviewer recorded the selected cards and summarized salient comments on a tally sheet. Informants were then asked to complete the card sort process a second time, in relation to their state of being *now *(after they had experienced a shift in well-being). Fifty-three cards reflecting positive states of well-being were presented. The second card sort process repeated the process used for the negative states. Interviews ranged between one and three hours in length.

Following the interview, the interviewer recorded the tally of all the cards endorsed, rejected, edited, and left as "not quite right" by the informant. These data were then computer-entered using a data entry program designed for this purpose. Once all interviews were completed, a tally was created from all participant responses summing how many individuals placed each item in the "applies," "best applies," "not quite right," or "does not apply" categories. The "applies" and "best applies" categories were subsequently combined to obtain a more stable metric. Tally results (see Table 4) were closely examined to identify those items that were consistently endorsed in positive and negative frames and thus were candidates for a directional scale item. A listing was also created that showed every item edit provided by participants. Thus the card sort process allowed us to quantify the level of endorsement for particular items among informants as well as to record comments and item edits, a process that guided the development of the final questionnaire.

#### Ib: Results -- Qualitative analysis

Qualitative analysis revealed that informants had a difficult time endorsing very negative items, for example, "I had lost my faith" or "I was hopeless." Informants explained that these items were "too absolute" and "too intense," and during their selection of cards they tended to offer explanations for why they did not feel comfortable choosing these terms. Most commonly, the explanation offered was that their situation "was bad--but not *that bad." *Their difficulty in selecting extremely negative anchor points led the research team to evaluate response formats which would allow informants to respond on a continuum instead of having to select among two extremes (see Phase Ic below).

Data analysis revealed that the majority of informants in this phase experienced the evocative card sort interview process as useful in understanding their own experience. Comments, which emerged organically at the close of the interview, reflected a range of insights including: "It got me thinking. I never thought about my experience like this before," "Now I understand how I got through this," and "I didn't know how far I had come." Querying how this process had occurred, several informants explained that sorting through the cards allowed them to verbalize feelings in a way that they had not done before, and that this afforded them a sense of clarity. Comments such as these were confirmation that the evocative card sort interview method had worked as intended and fostered reflexivity as well as enabled informants to put into words, states that had not previously been expressed.

#### Ib: Results -- Quantitative analysis

Table 4 shows the levels of endorsement for the items, divided by domain for ease of evaluation. The items were assigned domains subsequent to their identification through the qualitative process in Phase Ia; the uneven distribution is an outcome of the process and was not planned. Further, the high number of "whole person" statements reflects the nature of the qualitative data. Overall, fewer of the negative items were rated as "applies/best applies" than positive items, with negative items receiving on average 20 endorsements, and positive items 27, in spite of almost equal numbers of cards (54 and 53 respectively). For both positive and negative cards, about 13% were initially put into the "not quite right" stack, and of these, about 40% were modified to "applies." This occurred for two reasons. Some participants never were able to modify the card appropriately and eventually put the card in the "does not apply" pile. Others had found more appropriate cards later in the pile and no longer wanted to work with the "not quite right" item. Notably, the majority of completed edits for both positive and negative items were directed toward making them less absolute.

### Phase Ic: Developing draft instrument format

#### Measuring change

In parallel with the identification of item content, the research team considered the types of response options that that would be most appropriate for the assessment of patient-centered outcomes in the context of a clinical trial. We reviewed the ways in which different objective and subjective phenomena or attributes (e.g., frequency, duration, severity, satisfaction, agreement, or change) are commonly quantified through the use of response sets/scales [31]. However, while reading the study transcripts from which the item content was being derived, it became clear that the traditional response sets/scales applied in a standard clinical trial model relying on baseline (pre-test) and subsequent serial assessments (post-tests) would likely be problematic. Early in the phase Ia data analysis, the issue of "surprise" began to appear in the transcripts. This surprise was in relation to the nature of the experiences in relation to CAM therapies ("I never knew that I could feel like this") or in the extent of the change ("I never imagined that I could feel so much joy"). In this evaluative context, where change from baseline is the efficacy endpoint, frame or response shift can be a significant concern and a threat to internal validity. As defined by Sprangers and Schwartz [41], response shift is a change in the meaning of one's self-evaluation of the construct of interest (e.g., quality of life) as a result of: (1) a recalibration of the respondent's standards of measurement; (2) a change in the respondent's values; or (3) re-definition or re-conceptualization of the construct. To avoid the measurement error associated with response shift, we chose the evaluative methodology called the retrospective pretest, which has been suggested to be valid when the subjective experience of change is most salient [42].

#### The development of a response set format

At the end of the card sorts, we showed informants different possible question formats to identify those that resonated with the interviewees' issues. Response sets such as "never-always" or "strongly agree-strongly disagree" did not address the interviewees' needs for lessened intensity. As noted earlier, many informants commented during the evocative card sort that the descriptors were too intense, and they requested modifications that would soften the intensity of the meaning. To address these comments, and to meet our goal of providing positive as well as negative directions on the final instrument, the study team developed, and then piloted with participants, the approach of creating pairs of words that anchored two ends of the same continuum (e.g., "hopeless-hopeful"). The intent was to allow the respondents to choose where they fell on that continuum "before" the treatment or intervention and "now." Participants easily grasped how to work with these word pairs and indicated that this format addressed the issue of gradation of intensity. Thus we moved from the lists of descriptors evaluated in the evocative card sort interviews to word pairs. We were also sensitive to the time needed for participants to consider and respond to these items, and in order to minimize participant burden we chose to work toward a target length of 15 to 20 item pairs.

#### Creating and choosing word pairs for draft instrument

There were several steps in the process of draft instrument design. First, investigators utilized the ranked tallies (Table 4) to create word pairs that identified continua, attempting to capture a set of pairs that represented the most highly endorsed positive and negative items, to minimize redundancy when several items had similar meanings, and to cover the domains indicated in Table 4. Second, new tallies were run with participants subdivided into important categories, including race/ethnicity, type of CAM therapy (practitioner-based or self-practice), and gender. The aim was to examine whether our shortened list of pairs lacked any specific pairs that were preferentially endorsed by a single group as a crosscheck for important items that might have been missed in the tally approach. The investigators reviewed the new tallies and the draft item list to assure that the list did not omit any items that were particularly important to a group of respondents. Pairs were added as necessary to meet this criterion. Third, practitioners reviewed item pairs to assure that dimensions that were considered highly important within particular CAM therapies were not omitted. Some items in the physical domain were added back at this stage.

At the same time as instruments were being developed, we had opportunities to pilot draft instruments in two clinical trials and chose to do so with instruments developed up to that point. This process of finalizing instruments for RCTs provided additional feedback from the investigators and staff of these clinical trials, and from some participants in those studies who were asked for feedback. Further input was sought from colleagues interested in potentially using the instrument. By the end of the process, 18 pairs were chosen for further refinement via cognitive interview [43] testing. This draft instrument is shown in Table 6 by domains. The level of endorsement (rank) of the comparable card sort descriptors is also shown. The draft instrument was piloted in several test environments to establish how the measurement axis should be displayed, and what instructions were adequate. The environments included a graduate-level medical anthropology class, a class for students learning spiritual healing, a small clinical trial of tai chi for cancer patients, and parents participating in a healing touch camp for families caring for children with severe disabilities.

**Table 6:** Item Pairings and Domains for *Draft* Instrument for Cognitive Interviews Based on Rank () of Endorsement from Card Sort Interviews

Domain Negative Item	Item Pairing and Ranking		Domain Positive Item
Physical	***Not sleeping well***	***Sleeping well***	Physical
	***(6)***	***(10)***	

Physical	***Dull senses***	***Vibrant senses***	Physical
	***(7)***	***(7)***	

Physical	***Depleted***	***Vitalized***	Whole person
	***(4)***	***(6)***	

Affective	Suffering*	***Joyful***	Affective
	***--***	***(2)***	

Physical	***Exhausted***	***Energized***	Physical
	***(3)***	***(17)***	

Cognitive	Scattered*	***Focused***	Cognitive
	***--***	***(6)***	

Whole Person	Powerless*	***Empowered***	Whole person
	***--***	***(4)***	

Spiritual	***Hopeless***	***Hopeful***	Spiritual
	***(16)***	***(2)***	

Whole person	***Unforgiving***	***Forgiving***	Whole person
	***(18)***	***(3)***	

Social	Isolated*	***Connected***	Social
	***--***	***(5)***	

Whole person	Life has no meaning*	***Life has meaning***	Whole person
	***--***	***(3)***	

Spiritual	***Have no faith***	***Have faith***	Spiritual
	***--***	***(3)***	

Whole person	***Overwhelmed***	***Resilient***	Whole person
	***(4)***	***(4)***	

Affective	***Closed-hearted***	Open hearted**	Affective
	***--***	***(2)***	

Whole person	***Broken***	***Healed***	Spiritual
	***--***	***(2)***	

Whole person	***Defined by my illness or problems***	***Not Defined by my illness or problems***	Cognitive
	***(16)***	***(8)***	

Spiritual	***Not on a spiritual path***	***On a spiritual path***	Spiritual
	***--***	***(5)***	

Physical	***My body does not recover quickly***	***My body recovers quickly***	Physical
	***(15)***	***(1)***	

* Some items were not in the original data set and were created as new negative/positive pairing.

** In original items, "I am able to love" received 21 endorsements as "best applies" and "applies." Based on feedback from some early interviews this was changed to "closed-hearted" and "open-hearted" as many respondents in the interviews were unwilling to say that they were "unable to love." The domain and item endorsement for "open-hearted" corresponds to "I am able to love" in the original data set.

#### Instrument layout

The final draft instrument layout is shown in Table 7. The 100 mm blank line, without numbers or internal reference points, was the final consensus layout. Respondents indicate "before" and "now" on the same line; for data entry, the positions of the "before" and "after" points are measured as the distance in millimeters from the left edge of the line. We also successfully implemented this as a web-based data entry system in which the participants move a slider along the line to place the indicator.

**Table 7:** Sample Items Showing Pair Layout

**"B" is for where you were *before* <insert event here> began**. **"N" is for where you are *now***. **Place a "B" and an "N" based on how you see things now**.		
Not sleeping well	_________________________________________	Sleeping well

Dull senses	_________________________________________	Vibrant Senses

#### Further testing

In Phase II, reported in separate papers, the draft instrument was further evaluated following recommendations from the FDA guidance on developing PRO measures [31]. This included cognitive interviews [33,43], and quantitative evaluation in five different settings to check construct validity, the psychometric properties of the items and overall instrument, face validity in relation to different types of CAM therapies and ease of use by different populations [manuscript under development].

## Discussion

A growing body of literature reports that patients using CAM indicate experiencing shifts in well-being that extend beyond resolution of the symptoms from which the patient sought relief. These shifts include improvements in overall well-being, energy, clarity of thought, emotional, social, and physical functioning, control/empowerment, connection, and increased focus on one's inner life and spirituality [44-46]. However, the lack of appropriate tools to measure these emergent outcomes in a valid, reliable, comprehensive, and patient-centric way has limited the assessment of them. The two fully patient-centered instruments, the MyMOP and MyCAW [47,48], have patients identify their most important problems through open-ended questions, and rate their severity over multiple time points. These instruments, however, do not permit the interpretation of those metrics of change across studies, nor do they allow for the capture of unanticipated changes. Our team undertook a research program to develop and evaluate a patient-centered outcome measure to assess impacts of treatments within CAM systems of medicine. This outcome measure was developed through the use of a methodological approach that began not from existing constructs but rather by listening to the experiences of individuals who had undergone CAM treatment. To do so required development of a novel methodology to capture these sensitive shifts.

Our evocative interview and card sort process was innovative in several ways. Once participants selected items, they were asked how the item could be changed to better fit their experiences. In this way the card sort was flexible and responsive to participants' suggestions as they were encouraged to discuss, change and generate new items. The process of evocative interviewing appeared to be therapeutic, in that it provided participants an opportunity to talk about their personal experiences and understand them more deeply. Given time, a supportive environment, and the presence of research staff trained to be empathetic witnesses [49], many participants took the opportunity to tell their stories and to bring previously buried experiences to the surface. Many expressed gratitude after the interview for the opportunity to tell their stories and reflect on changes that had occurred in their lives. Some indicated that this was the first time they had spoken these stories and in the course of doing so gained insight into their own lives. Interviewers were commonly moved by the experience of witnessing the evocative interview process.

In the process of developing the measure, we struggled to find an appropriate way of presenting the word choices that informants had selected or adapted. In developing these word pairs, it was striking that domains that were highly endorsed as relevant in the positive state were not as highly endorsed when framed in the negative, and vice versa (see Table 4). For example in the spiritual domains, 21 participants endorsed the positive item "I am hopeful", whereas only 7 participants endorsed "I had no hope" as a negative item. This may be due to linguistic features of our wordings or to experiential shifts of the participants, such that they only recognized the issue of hope as it reappeared, rather than as something that was absent. Others have reported that negative items are predictive of different types of outcomes than positive items [50]. This is an area that we explore in our quantitative validation, and that would be appropriate for future qualitative and quantitative research with the instrument.

With regard to diversity in participant responses, it is noteworthy that in Phases Ia and Ib where metaphorical language and full narratives were analyzed, the researchers identified some minor gender, race/ethnicity, and CAM therapy differences in the types of events and situations that were reported as the source of their difficult situations. However, during our crosscheck of the card sort responses by participant category, few differences were identified in item endorsement frequencies. Thus, more general descriptors of the shifts in well-being appear to be more broadly understood and potentially generalizable, regardless of the source of an individual's difficulties.

Our limited sample size restricted our evaluation of diversity associated with different types of CAM therapies to two broad classes: therapies provided by practitioners (e.g. massage, TCM) and self-practices (e.g. yoga, meditation), and we were careful to include outcomes that were rated as relevant for both in our final list. However, in the psychometric evaluation, we hope to begin to explore whether different patterns of outcomes are associated with different CAM therapies. It seems probable that there will be differences associated with whole system interventions such as TCM and Ayurveda (which target many symptoms and conditions simultaneously) versus those interventions that only target specific symptoms (e.g. massage for low back pain), such as those reported by Hsu et al [12].

The content of our list of items to undergo further testing compares favorably with that presented in recent papers summarizing the qualitative research in CAM [45,46], and responds to the recent call for the development of such an instrument [51]. As we listened to the voices of our participants, and then developed the more streamlined language of the items, it because increasingly clear that the items set, or a subset of the items, may also be appropriate for use in other settings of complex interventions, such as cardiac rehabilitation, wellness and other lifestyle interventions, mental health interventions, or life coaching settings. It is our hope that this instrument might, as a whole or in part, move into the mainstream of patient-reported outcomes.

Our identification of the need for a retrospective assessment approach is consistent with the results in other fields [52-55]. This measurement problem has been shown to occur in some areas of education and program evaluation, where participants may indicate greater confidence in their knowledge of a topic before an educational session than after. This may be because their notion of how much there is to know has changed, or because their assessment of what they do know has changed, as a result of the session [53]. In these settings, it appears likely that the estimates of change are more accurate if the respondent rates both time points after the session, instead of having one rating before and the other after the intervention [56].

In relation to CAM research, meditation researchers have expressed concern that scales designed to evaluate participants' changing experiences of meditative states may not provide accurate change scores when administered pre and post. As individuals with no experience become novices and begin a meditative practice, the meanings of the words in the scales may change for them. And as novices become experts, their abilities to discern more subtle states are enhanced, leading to shifting response frames [57].

The types of biases that are usually associated with standard pre and post measurement of change and with retrospective pretest measurement differ, and it is rare to find settings where the two approaches to assessing patients' subjective states can be compared with a biomarker that can be used as a gold standard. However, in 2007, Nieuwkerk et al. identified such an opportunity in their study of fatigue among patients with HIV infection [56]. In a longitudinal assessment of changes in fatigue levels and quality of life, they found that the retrospective pretest approach to measuring change in fatigue and well-being was more highly correlated with changing viral loads than were contemporaneous assessments. The authors attribute this to a changing internal baseline, such that patients who are worsening may not have a good idea of the full range of possibilities at the initial time points. This has been seen in relation to worsening in other conditions as well [58]. CAM interventions appear from our data to be associated with changing internal baselines in relation to improvement. Thus for CAM interventions, we view the retrospective pretest as a viable option in the assessment of subjective shifts in well-being, and this approach is further evaluated [manuscript in development].

### Study limitations to this point

Although our base sample of 119 individuals providing qualitative interviews for secondary analysis was substantial, our study sample for subsequent item development and testing has been relatively small. Phase II, including 28 participants in cognitive interviews [59] and more than 600 participants completing the draft instrument [manuscript in development], provides greater diversity in gender, race/ethnicity, and education. Phase II also provides greater diversity in the types of conditions being addressed, as well as types of CAM therapies utilized, and will permit us to evaluate the range of responses per item, full use of the scale, and other features of response. Items at this point in the development process were chosen to cover the breadth of experience reported by our informants. The psychometric assessment will provide guidance as to the level of inter-correlation among the items, and any scaling embedded within the instrument. Further, the psychometric assessment will allow the measurement of construct validity for items, such as depression and sleep, against validated scales.

## Conclusions

Our research team sought to develop an instrument to document CAM patients' complex shifts in well-being by adopting a methodological approach that began not from existing constructs but rather by listening to the experiences of individuals who had undergone CAM treatments. We then built upon patient reports of subjective shifts in well-being associated with these therapies with the aim of establishing a reasonably small set of items that were faithful to the patient narratives and covered their most salient changes.

This paper reports the success of a novel approach to the development of outcome instruments. Overall, while our samples size to develop items was relatively large, our sample size used to determine the item list was relatively small. However, our cognitive interviews, presented in a companion paper [59], have contributed substantially to the effort to refine the questionnaire by identifying word pairs that are clear and understood similarly across participants, and are viewed by participants as representing positive and negative endpoints of the same conceptual/experiential continuum. Our validation process (manuscript in preparation) indicates that participants are willing to use the full scale, and are willing to report shifts in the negative as well as positive direction. Data collected on groups of subjects varying by the types of interventions they experienced also suggest that different interventions may be associated with different characteristic patterns of change.

The instrument is undergoing continued testing, and is available for use by cooperating investigators. We look forward to continuing development and testing of this tool, and welcome collaborators who would like to work with it and to share their experiences as well as their anonymized data with us. The final version is available at our website, http://www.selfassessmentofchange.org.

## Competing interests

The authors declare that they have no competing interests.

## Authors' contributions

CR was the PI of the project, oversaw all aspects related to this paper, and led the writing of the paper. MN (Mimi) served as qualitative methodologist for phases1a and 1b, and led the writing of section 1b. MN (Mark) provided overall theoretical and methodological guidance and critical support for all aspects of the paper. KK served as project manager for Phase 1b, conducted interviews during Phase 1b, assisted in qualitative analysis and supported all aspects of manuscript writing and completion. CS served as co-investigator, provided critical input throughout project phases 1a and 1b, and conducted interviews in Tucson and Portland. IB actively participated throughout phases 1a and 1b, and contributed a project data set to phase 1a. HC served as project manager for Phase 1a and provided critical support in manuscript development for that phase. CE contributed one of the project data sets to phase 1a, and provided support for data analysis and interpretation. MK actively participated in all data analysis and reduction for phases 1a and 1b, and provided data to phase 1a. ES contributed project data, coding, analysis, and interpretation in phase 1a, and provided critical input throughout phase 1b. MV provided two data sets for phase 1a, supervised coders for phase 1a, provided and supervised an interviewer in Phase 1b, and was actively engaged in all manuscript preparation activities. SW provided a data set and coder to phase 1a, and was actively involved as an investigator. SJC served as the team psychometrician, providing guidance on the development of PROs, the development of the retrospective pretest approach, format, and metrics, and all aspects of paper writing. All authors contributed to the final manuscript and approved it.

## Pre-publication history

The pre-publication history for this paper can be accessed here:

http://www.biomedcentral.com/1472-6882/11/135/prepub
